# Contrast enhanced magnetic resonance imaging highlights neurovasculature changes following experimental traumatic brain injury in the rat

**DOI:** 10.1038/s41598-020-77975-2

**Published:** 2020-12-04

**Authors:** David K. Wright, Jamie N. Mayo, Mujun Sun, Terence J. O’Brien, Sandy R. Shultz

**Affiliations:** 1 Department of Neuroscience, Central Clinical School, Monash University, The Alfred Centre, 99 Commercial Road, Melbourne, VIC 3004 Australia; 2 Department of Medicine, Royal Melbourne Hospital, University of Melbourne, Parkville, 3052 Australia; 3 IDEAS 2.0 Center of Innovation, VA Salt Lake City Health Care System, Salt Lake City, UT USA; 4 Division of Epidemiology, Department of Medicine, University of Utah School of Medicine, Salt Lake City, UT USA

**Keywords:** Experimental models of disease, Preclinical research, Brain injuries, Neurovascular disorders

## Abstract

Neurovascular injury has been proposed as a universal pathological hallmark of traumatic brain injury (TBI) with molecular markers of angiogenesis and endothelial function associated with injury severity and morbidity. Sex differences in the neurovasculature response post-TBI may contribute to the differences seen in how males and females respond to injury. Steady-state contrast enhanced magnetic resonance imaging (SSCE-MRI) can be used to non-invasively assess the neurovasculature and may be a useful tool in understanding and predicting outcomes post-TBI. Here we used SSCE-MRI to investigate the neurovasculature of male and female rats at 48 h after an experimental TBI, and how these changes related to neuromotor function at 1-week post-TBI. In addition to TBI induced changes, we found that female rats had greater vessel density, greater cerebral blood volumes and performed better on a neuromotor task than their male counterparts. These results suggest that acute post-TBI cerebrovascular function is worse in males, and that this may contribute to the greater functional deficits observed post-injury. Furthermore, these results highlight the potential of SSCE-MRI to provide insights into the cerebral microvasculature post-TBI. Future studies, incorporating both males and females, are warranted to investigate the evolution of these changes and the underlying mechanisms.

## Introduction

Neurovascular injury has been proposed as a universal pathological hallmark of traumatic brain injury (TBI)^1^. Changes in microvascular density have been detected acutely after TBI and are thought to contribute to early pathogenesis and reflect the extent of neurological damage^2,3^. Microvascular density in the weeks following TBI has also been associated with recovery^3,4^, and alterations in cerebral blood flow can persist for months post-injury and may contribute to both recovery and continued pathology^3,5^. Molecular markers of new blood vessel growth, angiogenesis, and endothelial function have also been associated with injury severity and morbidity after TBI^6^.

There is increasing evidence that males and females differ in various outcomes after TBI, underscoring the notion that there are biological differences between the sexes that account for this^7–11^. Of particular relevance, there is initial evidence suggesting sex differences in neurovascular measures post-TBI, and that these measures correlate with neurological recovery^3,12^. For example, a recent study found that there were similar vessel losses in both males and females at 1-day post-injury, but revascularization was greater and more complex in the ipsilateral cortex of males at 7 days post-injury^4^. Given the role of the neurovasculature in both acute and long-term consequences of TBI, and the association of neurovascular response with TBI severity and morbidity, acute vascular density changes could be an important sex specific prognostic indicator of patient recovery.

Identifying clinically applicable biomarkers that are sensitive to sex differences would aid in the development of personalized therapeutic approaches for the treatment of TBI. Steady-state contrast enhanced magnetic resonance imaging (SSCE-MRI) is a relatively recent imaging method that can be used to assess the neurovasculature^13^. SSCE-MRI uses an iron-based contrast agent and allows the estimation of cerebral blood volume (CBV) changes in the microvasculature, as well as changes in vessel density and size. Importantly, SSCE-MRI derived measurements of vessel density and size have a strong positive correlation with histological measurements^13,14^, and the technique has been utilized to monitor the treatment effect of drugs targeting angiogenesis^15^.

While SCCE-MRI provides a means to investigate sex-specific neurovascular changes after TBI, its utility in this context has yet to be explored. Furthermore, it is unknown how SSCE-MRI measures relate to recovery after TBI. Therefore, this study used a pre-clinical rodent model of TBI to test the hypothesis that SSCE-MRI neurovasculature measurements could identify sex differences post-TBI and examined how SSCE-MRI measures related to functional outcomes.

## Materials and methods

### Subjects

51 adult male (n = 26) and female (n = 25) Long-Evans rats were purchased from Monash animal research services (Melbourne, Australia). All rats were 10–15 weeks of age at the time of surgery with male rats significantly heavier than female rats (Table 1). All females were in diestrus at the time of injury. Following surgery, rats were housed individually under a 12 h/12 h light/dark cycle with ad libitum access to water and food for the duration of the study. All experimental procedures were approved by The University of Melbourne and The Florey Institute of Neuroscience and Mental Health animal ethics committees, and complied with the guidelines of the Australian Code of Practice for the Care and Use of Animals for Scientific Purposes.

**Table 1:** Acute injury severity measures and cohort numbers.

Cohort	Male + Sham	Male + TBI	Female + Sham	Female + TBI
n	11	15	11	14
Weight (g)	463.7 ± 62.0	450.1 ± 41.6	291.6 ± 9.4	302.5 ± 25.9
Pain reflex (s)	0	269.5 ± 94.8	0	355.3 ± 126.6
Self-righting (s)	292.1 ± 68.9	844.6 ± 266.4	154.6 ± 54.7	935.0 ± 276.3

Male rats weighed significantly greater than female rats at the time of injury (*p* < 0.0001). Rats given a TBI had significantly longer pain reflex and self-righting times compared to sham rats (*p* < 0.0001). Numbers represent mean ± SD.

### Experimental design

Rats were randomly assigned to either sham (n = 11 male; n = 11 female) or lateral fluid percussion injury (LFPI) groups (n = 15 male; n = 14 female) and underwent 48-h SSCE-MRI (n = 12 male, 6 LFPI; n = 12 female, 6 LFPI) and/or 1-week behaviour (n = 23 male, 14 LFPI; n = 19 female, 10 LFPI). Animal numbers were determined based on earlier SSCE-MRI and behaviour studies^16,17^ and animals were randomly assigned to each group.

### Lateral fluid percussion injury (LFPI)

Sham and LFPIs were performed as described previously^18–20^. Briefly, rats were anaesthetized and their head was immobilized using a standard stereotaxic device with anesthesia maintained using 2% Isoflurane in 500 ml/min oxygen via a nosecone. The dura matter of the left hemisphere was exposed by a craniectomy (5 mm diameter) centered -3.0 mm posterior and 4.0 mm lateral to bregma. A hollow injury cap was fixed to the skull, anesthesia was terminated, and the injury cap connected to the fluid percussion device. At the first sign of hind-limb withdrawal to a toe-pinch, a fluid percussion pulse of 3 atmospheres was delivered to the intact dura. Pain (i.e. hind-limb withdrawal to a toe-pinch) and self-righting reflexes were recorded immediately post-injury (see Table 1)^21^. Once spontaneous breathing had resumed the injury cap was removed and the incisions sutured. Sham rats underwent the same procedure, including craniectomy, but no fluid pulse was given.

### Steady-state contrast enhanced MRI (SSCE-MRI)

SSCE-MRI was performed using a 4.7 T MRI at 48 h post-injury. Baseline T_2_^*^-weighted and T_2_-weighted images were acquired over the same 12 slices with a field of view = 38.4 × 38.4 mm^2^ and slice thickness = 1 mm. T_2_^*^-weighted images were acquired with a FLASH sequence with repetition time (TR)/echo time (TE) = 400/10 ms; number of excitations (NEX) = 6; and flip angle = 20°. T_2_-weighted images were acquired with a RARE sequence and TR/TE_eff_ = 2300/65 ms; RARE factor = 8; and NEX = 8. Following the acquisition of these preliminary images, a bolus of 15 mg/kg of ferumoxytol (Feraheme, AMAG Pharmaceuticals, Lexington, MA) was delivered via a tail vein cannula and both T_2_^*^-weighted and T_2_-weighted image acquisitions were repeated 5 min later.

### Image analysis

Vessel density, vessel size, ΔR_2_ (CBV in small blood vessels) and ΔR_2_^*^ (CBV in a broad range of vessels) images were calculated using MATLAB (R2018b, The MathWorks, Natick, MA) with formulae as described in Lin et al.^13,22^. These transverse relaxation rate changes were then used used to investigate vessel size (ΔR_2_^*^/ΔR_2_) and density (ΔR_2_/(ΔR_2_^*^)^2/3^)^23,24^. Regions of interest (ROI) were delineated in the ipsilateral and contralateral cortex and hippocampus and the median value for each image metric determined.

### Neuromotor testing

Neuromotor function was assessed one-week post-injury using a 1 m long, 2 cm wide elevated wooden beam as previously described^17,21^. Briefly, rats were trained on the beam task on the day prior to testing. On the day of testing, rats completed ten trials with the time taken to traverse the beam, as well as the numbers of slips and falls, recorded. The maximum time allowed per trial was 60 s, which was the value given to rats that fell.

### Statistical analysis

Two-way analysis of variance (ANOVA), with sex and injury as between-subjects factors, was used for the statistical analysis of all variables SPSS 23.0 (IBM Corp., Armonk, USA). Bonferroni post-hoc comparisons were completed when appropriate. Statistical significance was set at *p* < 0.05.

## Results

### Sex and TBI affect SSCE-MRI measures

SSCE-MRI was used to study the vasculature of brain tissue 48 h after TBI or sham injury in male and female rats. Measures of vascular density, vessel size, ΔR_2_^*^ (i.e. CBV changes in a broad range of vessel sizes), and ΔR_2_ (i.e. CBV changes primarily in small vessels such as capillaries and venules) were evaluated in the cortex and hippocampus both ipsilateral and contralateral to the injury site.

**Figure 1:**
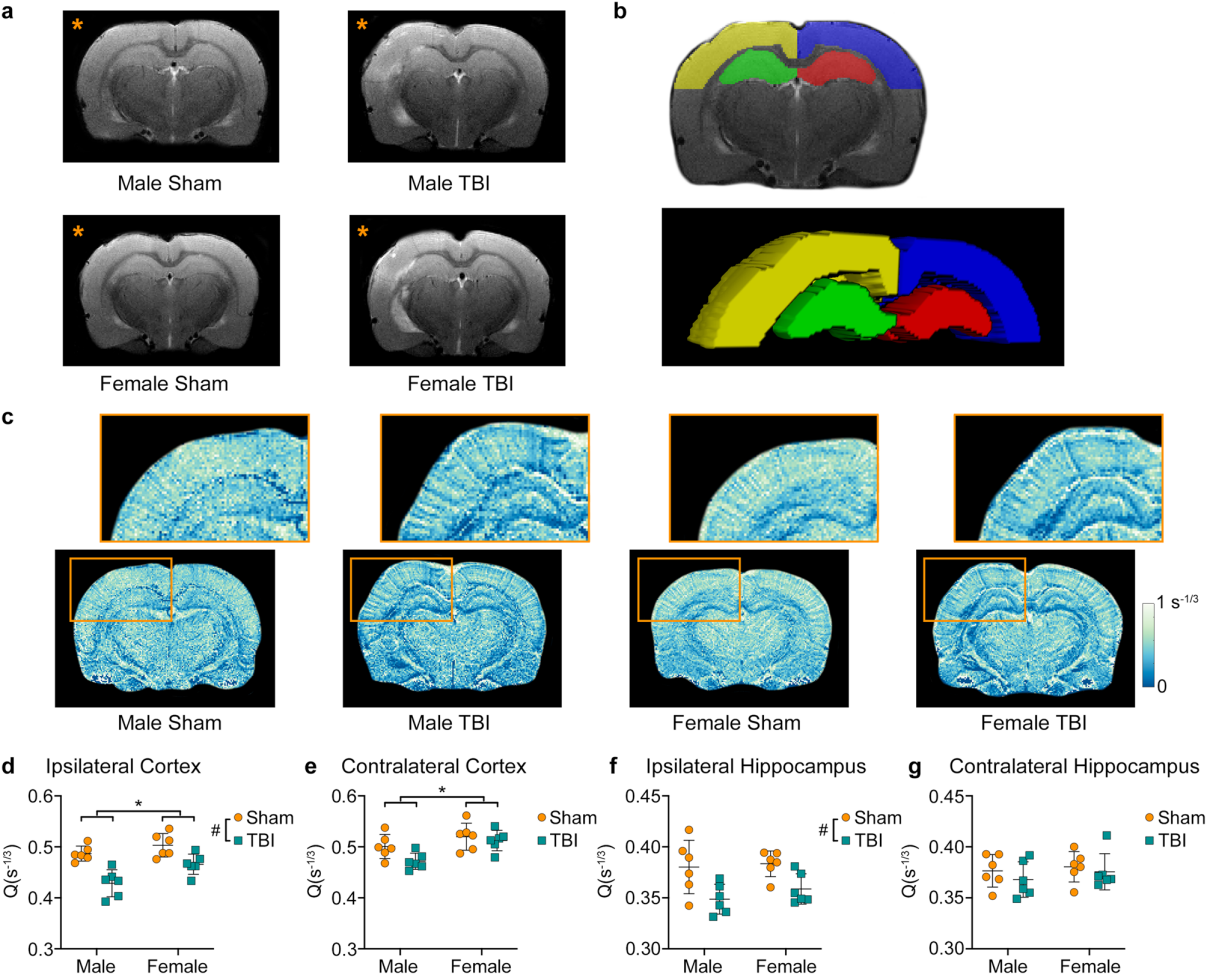
TBI results in decreased vascular density (Q). (**a**) Representative T2-weighted images from male sham, male TBI, female sham, and female TBI rats showing the extent of injury in the ipsilateral hemisphere (orange*). (**b**) Example ROIs for one animal: yellow, ipsilateral cortex; green, ipsilateral hippocampus; blue, contralateral cortex; and red, contralateral hippocampus. (**c**) Representative Q images from male sham, male TBI, female sham and female TBI rats. The ipsilateral hippocampus and cortex are shown enlarged above (orange box). Region-based analysis revealed a significant main effect of sex in both the (**d**) ipsilateral and (**e**) contralateral cortices with females having greater Q than males. Furthermore, there was a significant injury effect with TBI rats having reduced Q in the ipsilateral (**d**) cortex and (**f**) hippocampus. Graphs show mean + SD. * Significant (*p* < 0.05) differences between males and females; # significant (*p* < 0.05) differences between sham and TBI.

Representative T2-weighted images show the effects of LFPI with evidence of edema and white matter damage (Fig. 1a). Example ROIs for one rat are shown in Fig. 1b. Representative images of vascular density are shown in Fig. 1c. Two-way ANOVA found a significant main effect of injury in the ipsilateral cortex (F_1,20_ = 29.666, *p* < 0.001, Fig. 1d) and ipsilateral hippocampus (F_1,20_ = 14.825, *p* = 0.001, Fig. 1f), with TBI rats having less density. There was also a significant main effect of sex in the ipsilateral cortex (F_1,20_ = 9.373, *p* = 0.006, Fig. 1d) and contralateral cortex (F_1,20_ = 11.323, *p* = 0.003, Fig. 1e), with female rats having greater density than males. No significant differences were observed in the contralateral hippocampus (Fig. 1g).

**Figure 2:**
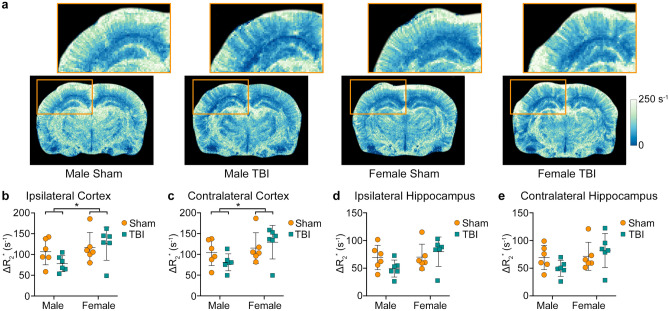
SSCE-MRI revealed sex differences in blood volume. (**a**) Representative images showing increased ΔR_2_^*^ values in females compared to males. The ipsilateral hippocampus and cortex are shown enlarged above (orange box). Region-based analyses revealed that male rats have reduced blood volume, as indicated by lower ΔR_2_^*^ values, in (**b**) ipsilateral and (**c**) contralateral cortices. Graphs show mean + SD. * Significant (*p* < 0.05) differences between males and females.

Figure 2 shows representative images of ΔR_2_^*^. Two-way ANOVA found a significant main effect of sex in the ipsilateral cortex (F_1,20_ = 4.766, *p* = 0.041, Fig. 2b) and contralateral cortex (F_1,20_ = 4.820, *p* = 0.040, Fig. 2c), with female rats having greater ΔR_2_^*^ than males. There were no significant differences observed for either the ipsilateral or the contralateral hippocampus (Fig. 2d,e, respectively) and no significant effect of injury was observed in any of the ROIs investigated.

**Figure 3:**
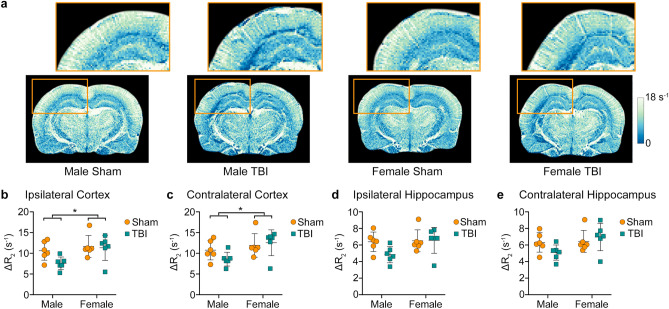
SSCE-MRI revealed sex differences in the blood volume of smaller vessels such as capillaries and venules. (**a**) Representative images showing increased ΔR_2_ values in females compared to males. The ipsilateral hippocampus and cortex are shown enlarged above (orange box). Region-based analyses revealed that male rats have reduced blood volume in smaller vessels, as indicated by lower ΔR_2_ values, in (**b**) ipsilateral and (**c**) contralateral cortices. Graphs show mean + SD. *Significant (*p* < 0.05) differences between males and females.

Representative images of ΔR_2_ are shown in Fig. 3. There was a significant main effect of sex in the ipsilateral cortex (F_1,20_ = 5.762, *p* = 0.026, Fig. 3b) and contralateral cortex (F_1,20_ = 5.955, *p* = 0.024, Fig. 3c), with female rats having greater ΔR_2_ values than males. No significant differences were observed in either hippocampus (Fig. 1d,e) and no significant effect of injury was observed in any of the ROIs investigated.

There were so statistically significant differences found on the measure of vessel size (*p* > 0.050, data not shown).

### Males given TBI have worse neuromotor outcomes than females

**Figure 4:**
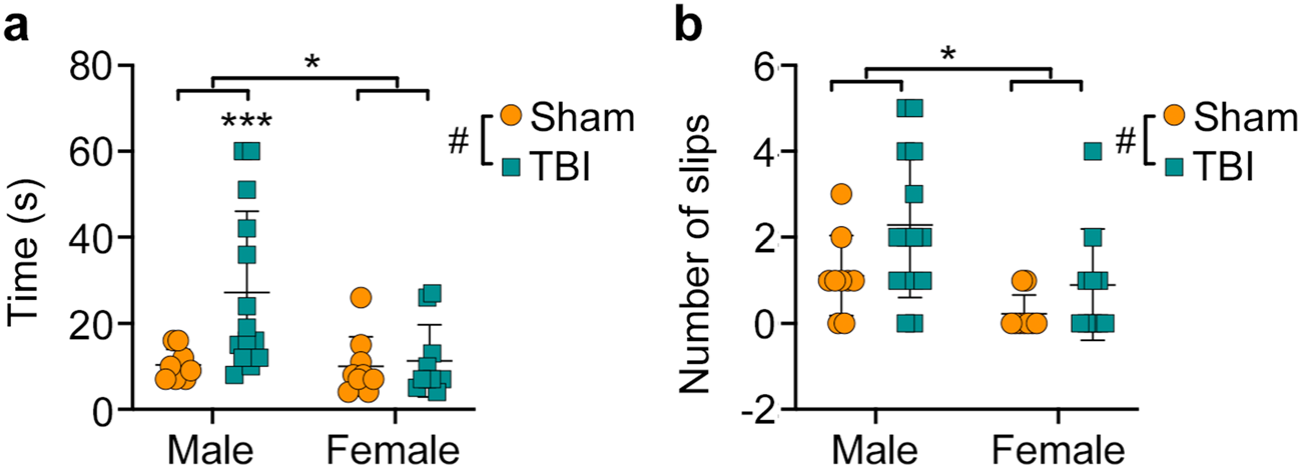
Behavioural testing revealed sex-specific deficits in sensorimotor function. (**a**) Male rats given a TBI had significantly longer traverse times than all other groups. There were also significant effects of injury and sex on traverse times. (**b**) Male rats and TBI cohorts made significantly more foot slips than female rats and sham cohorts, respectively. Graphs show mean + SD. ***p < 0.005; *Significant (*p* < 0.05) differences between males and females; ^#^Significant (*p* < 0.05) differences between sham and TBI.

Rats were tested on the beam task one-week post-injury to assess sensorimotor function. On the measure of traverse time, two-way ANOVA found a significant sex*injury interaction (F_1,38_ = 4.086, *p* = 0.049, Fig. 4a) with the Male + TBI rats having longer traverse times compared to each of the other groups (all *p* < 0.005). There were also significant injury (F_1,38_ = 5.562, *p* = 0.024) and sex (F_1,38_ = 4.442, *p* = 0.042) effects on the measure of traverse time. On the measure of slips and falls, two-way ANOVA found significant main effects of injury (F_1,38_ = 5.502, *p* = 0.024, Fig. 4b) and sex (F_1,38_ = 8.296, *p* = 0.006, Fig. 4b), with TBI and male rats having more slips.

## Discussion

The majority of experimental TBI research has been conducted in males, creating a void with respect to understanding how the female brain responds to TBI. The cerebrovasculature likely plays a key role in TBI recovery as it supports tissue metabolism, edema resolution, and the clearance of debris^4^. Here, we employed SSCE-MRI to interrogate the microvasculature of male and female rats given experimental TBI at 48 h post-injury, and how SSCE-MRI measures relate to neuromotor function at 1 week-post injury. We found that TBI decreased vessel density in both males and females. However, females had superior vessel density and CBV compared to males, and female rats given a TBI had better neuromotor function compared to male TBI rats.

Our results concur with earlier studies showing an acute reduction in vascular density followed by increased vascular structure at 1 week and 2 weeks post-injury^2,25^. Park and colleagues used a silicone microangiography technique and immunohistochemistry in male rats also given a 3 atm LFPI. The authors found a significant reduction in microvasculature density, with reduced capillary numbers and diameters at 24 h that had recovered by 2 weeks post-injury^26^. Obenaus and colleagues (2017) also reported a reduced neurovascular network within male rats one day after being given a controlled cortical impact injury. Furthermore, confocal microscopy findings suggested that reductions in density were due to increased fragmentation of the vasculature whereby smaller branching vessels disappear and larger vessels remain^27^. These results would align with changes in SSCE-MRI ΔR_2_ values following TBI, as they reflect CBV changes in smaller blood vessels. However, we did not find an injury effect in this study, possibly due to the timing or sensitivity of SSCE-MRI. Immonen and colleagues (2010) showed that after an initial decrease in ΔR_2_ values at the site of the lesion 1 to 4 h post-CCI injury, ΔR_2_ values had returned to sham levels by 48 h post-injury^16^. A subsequent study by the same group measured ΔR_2_ values 48 h post-LFPI and found no significant differences in CBV between sham and TBI rats which is also consistent with the results observed here^25^.

We found that female rats had greater vessel density, and higher ΔR_2_ and ΔR_2_^*^ values in the ipsilateral and contralateral cortex when compared to their male counterparts. As disruption to the cerebral vasculature can lead to secondary injury mechanisms including hemorrhage, edema, abnormal perfusion and blood–brain barrier dysfunction^28^ these results may in part explain the more severe sensorimotor deficits seen in male rats. Despite conflicting evidence on gender-specific outcomes, it is clear that sex plays an important role in TBI outcomes^11,29^. Notably, our sex-difference findings on the beam task are consistent with those of Wagner and colleagues (2004) who found that male rats given a controlled cortical impact TBI performed worse than females on a beam-walking task^30^.

There are some limitations to consider with the current study. We performed SSCE-MRI at a single acute time-point, and future studies should employ serial SSCE-MRI at acute, sub-acute, and chronic recovery times to investigate evolving changes. Our small cohort sizes for SSME-MRI coupled with our limited behavioural testing prevented us from conducting meaningful analyses to assess how the SSME-MRI changes relate to functional outcomes, which would be of great interest and should be explored further. We did not perform histological or immunohistochemical validation of altered vasculature and future studies could also complement SSCE-MRI findings with direct measures of cellular and molecular changes related to brain vasculature. For example, vascular endothelial growth factor (VEGF) is a potent stimulator of angiogenesis and vasculogenesis, and has been found to be upregulated in experimental TBI^28,31,32^. Another protein that can also stimulate angiogenesis is pleiotrophin which is upregulated in the ischemic brain, and of particular relevance here, in the endothelial cells of newly formed vessels^33^.

In conclusion, this study used SSCE-MRI to investigate the neurovasculature of male and female rats at 48 h after TBI, and how these changes related to neuromotor function at 1-week post-TBI. Although TBI decreased vessel density in both sexes, female rats had greater vessel density, ΔR_2_, and ΔR_2_^*^ values, and female TBI rats performed better on a neuromotor task than their male counterparts. These results suggest that cerebrovascular function, as assessed by SSCE-MRI, is superior in females and may contribute to their improved neuromotor recovery after TBI. We have also shown the potential for SSCE-MRI as a biomarker to provide insights into the cerebral microvasculature post-TBI. In practice, Ferumoxytol is a superparamagnetic iron oxide nanoparticle that was approved for the treatment of iron deficiency and anemia in patients with chronic kidney disease by the FDA. It has since been used for SSCE-MRI^34^, and has been proven safe and effective for clinical MRI use^35^. As such, SSCE-MRI could be a promising tool in detecting vascular density changes in humans with TBI. Finally, our results demonstrate the need for future translational TBI studies to incorporate both sexes, and to investigate how the neurovasculature evolves throughout TBI recovery. Such studies may provide a foundation to develop optimal interventions that improve recovery in both males and females.

## Data Availability

The imaging datasets analysed during the current study are available from the corresponding author on reasonable request.
